# In Memoriam: Julia L. Cook, PhD

**DOI:** 10.31486/toj.24.5048

**Published:** 2024

**Authors:** Richard N. Re

**Affiliations:** Scientific Director, Ochsner Clinic Foundation, New Orleans, LA

**Figure:**
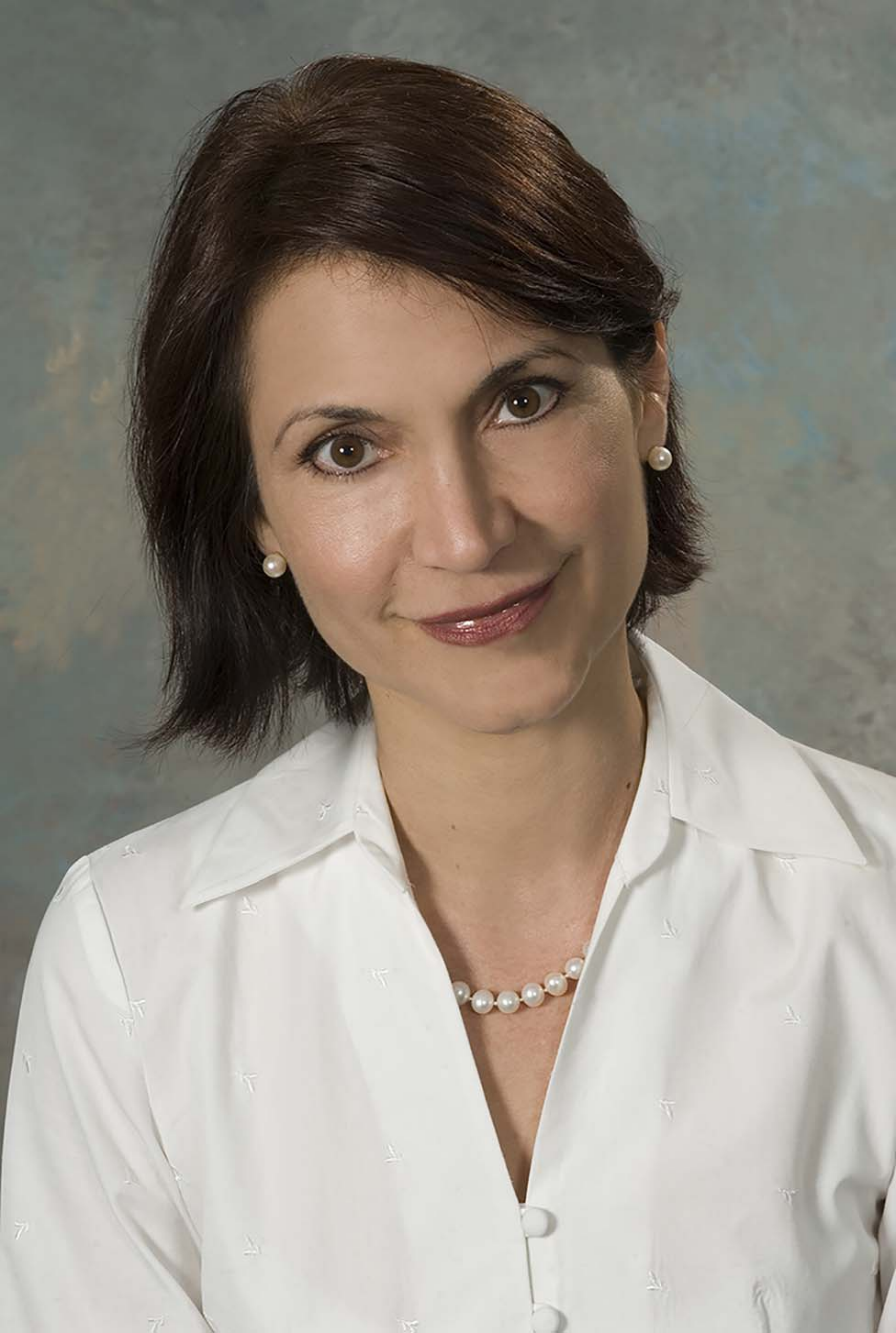
**Julia L. Cook, PhD** May 28, 1958 - August 26, 2024

Julia L. Cook, PhD passed away peacefully surrounded by her family on August 26, 2024, after a long battle with cancer. She was a remarkable person, respected and admired by all who knew her, a role model and mentor, a devoted friend, and a trusted confidant to so many. She also was an extraordinarily gifted scientist.

After graduating from North Carolina State University with a degree in genetics and completing postdoctoral training, she assumed the role of Director of Molecular Genetics in the Ochsner Research Division. It was then that I had the opportunity to collaborate with her. Having become interested in the intracellular action of certain peptide hormones (“intracrines”), I was looking for a colleague to aid in developing this idea. Julia was more than willing to help in spite of the other projects she had ongoing. Her work was simply first rate, helping to establish and define the intracellular actions of the peptide hormone angiotensin II in great detail. This work led to numerous papers in top-flight journals and to NIH funding. The details of angiotensin trafficking from the extracellular space to the cell nucleus, delineation of the second messengers activated by intracellular angiotensin II, the pathological role of angiotensin receptor (AT1R) fragments, the regulation of AT1R intracellular trafficking to the cell surface by a previously unrecognized associated binding protein (GABARAP), and the demonstration that the down regulation of that binding protein resulted in blood pressure lowering in hypertensive animals were only some of the results of her work.

Enabling the investigation of triplex DNA binding to inhibit cancer cell proliferation and the inhibition of erythroleukemia cell growth by triplex-forming RNAs are other examples of her wide-ranging capabilities. She designed and produced a fluorescent angiotensin II fusion protein that we not only used in a transgenic mouse model to demonstrate the pathological effects of overexpression of intracellular angiotensin II, but Julia also made the protein freely available to hypertension investigators around the country who, in turn, used it to make important breakthroughs such as demonstrating the role of renal tubular intracellular angiotensin II in hypertension. In many of these studies, her husband Dr Jawed Alam, who led his own research group and is a first-rate molecular geneticist himself, pitched in to help and thereby round out a very productive team. Later in her career, as Ochsner emphasized clinical research, Julia assumed the role of Administrative Director of the Institute for Clinical Research, managing research clinical trials. Again she excelled.

As productive as she was at her work, Julia had an even greater impact on those who knew her. She approached life as she approached science—with enthusiasm, skepticism, creativity, and awe. These traits came across whether she was talking about an experiment, reviewing a paper for the *Ochsner Journal* (for which she was an editorial board member), planning a social event, or providing advice to a friend. She was a loving mother to Lina and wife to Jawed. She and Jawed enjoyed life in a way that was contagious. Julia Cook was an extraordinary person on so many levels. I consider myself lucky to have known her. She will be missed.

